# Clinical and Genomic Features and Prognostic Biomarkers of Oligometastatic Nonsmall Cell Lung Cancer

**DOI:** 10.1016/j.cllc.2025.07.010

**Published:** 2025-07-31

**Authors:** Rebecca A. Deek, Jongmyung Kim, Ritesh Kumar, Benjamin Medina, John Langenfeld, Ioannis Kontopidis, Richard Lazzaro, Ke Nie, Lara Hathout, Ozan Guler, Cem Onal, Eshan Patel, Missak Haigentz, Salma K. Jabbour, Matthew P. Deek

**Affiliations:** 1 Department of Biostatistics, University of Pittsburgh, Pittsburg, PA; 2 Department of Radiation Oncology, Rutgers Cancer Institute of New Jersey, Rutgers Robert Wood Johnson Medical School, Rutgers University, New Brunswick, NJ; 3 Department of Surgery, Rutgers Cancer Institute of New Jersey, Rutgers Robert Wood Johnson Medical School, Rutgers University, New Brunswick, NJ; 4 Department of Radiation Oncology, Baskent University, Ankara, Turkey; 5 Division of Medical Oncology, Rutgers Cancer Institute of New Jersey, Rutgers Robert Wood Johnson Medical School, Rutgers University, New Brunswick, NJ

## Abstract

Metastatic non small cell lung cancer (NSCLC) is a heterogenous disease process. Here we show genomic alteraions correlate with disease volume in metastatic NSCLC. This may aid in disease prognostication in the future.

**Introduction:**

Treatment of metastatic disease is rapidly evolving with local therapies increasingly being used in patients with oligometastasis. Better personalization is needed to appropriate select patients for these interventions. We attempted to better understand clinical features and genomic features of oligometastatic nonsmall cell lung cancer (NSCLC).

**Materials and Methods:**

Patients with metastatic NSCLC were included and underwent next generation sequencing. Patients were defined as having either oligometastatic (≤5 lesions) or polymetastatic (≥ 6 lesions) disease. Median overall survival (mOS) was computed using the Kaplan Meier method and multivariable Cox regression (MVA) analyses performed. DAVID and PANTHER analysis identified pathways differing between oligo and polymetastasis.

**Results:**

A total of 406 patients were included. Oligometastasis was associated with improved mOS compared to polymetastasis (25.9 months vs. 18.7 months, *P* = .02) and remained so on MVA (HR 0.60, *P <* .001). Genetic features differed between oligometastatic and polymetastatic NSCLC. Oligometastatic patients had higher incidence of alterations in *PI3K* pathway genes (24.3% vs. 14.7%, *P* = .03) and *LRPB1* (7.8% vs. 2.5%, *P* = .03). while polymetastatic patients had higher incidence of mutations in *EGFR* (33.1% vs. 21.4%, *P* = .01) and *ALK* (8.6% vs. 3.3%, *P* = .03). On DAVID analysis, pathways important in motility and epithelial mesenchymal transition, including WNT and TGFB, were enriched in patients with polymetastasis.

**Conclusions:**

Oligometastasis is associated with improved prognosis. The underlying genetic composition differs between oligo and polymetastasis and might aid in better defining metastatic disease with loco-regional behaviors.

## Background

Metastasis has long been viewed as a binary process (presence vs. absence) and in turn historically limiting treatment to systemic therapy. Recent evidence now suggests metastasis is a heterogenous disease process likely represented as a spectrum of disease states.^1^ One point along this spectrum is oligometastasis, typically defined as ≤3-5 metastases, for which the few number of metastatic lesions is felt to represent a disease biology behaving more akin to loco-regional rather than widely systemic disease.^2^

An implication of an oligometastatic state characterized by an attenuated metastatic phenotype is that local therapies, such as radiation or surgery, to these lesions might result in prolonged disease-free intervals, and in some cases, even cure. To examine this hypothesis a series of prospective trials within lung, prostate, and metachronous disease of varying histologies tested the efficacy of metastasis directed therapy, typically using stereotactic ablative radiation (SABR), and demonstrated improvements in progression free survival (PFS) and overall survival (OS) with use of local therapy.^3-11^

While emerging clinical evidence suggests a benefit to metastasis directed therapy (MDT) exists, significant aspects of oligometastasis remain poorly understood, first amongst which is a biological understanding of the oligometastatic state. Current definitions rely on lesion enumeration, which while probably correlated with disease behavior, likely inadequately captures the heterogeneity of the metastatic spectrum. To this point, within metastatic castration sensitive prostate cancer, underlying genetic differences exist across the spectrum of metastasis and potentially provide superior prognostic discrimination compared to clinical features.^12,13^ In comparison, within nonsmall cell lung cancer (NSCLC), little is known regarding the genetic variability across the metastatic spectrum. Therefore, the goal of this study is to better characterize the clinical and molecular landscape of oligometastatic NSCLC within the broader spectrum of metastatic NSCLC to allow for more biological definitions of this disease state in order to understand its clinical behavior as well as drivers of cancer invasion and metastasis.

## Material and Methods

This study included patient with histologically confirmed metastatic nonsmall cell lung cancer from a single institution. Patients underwent next generation sequencing using a 324 gene panel (Foundation 1 CDx). Biopsy specimens from which NGS were performed were from either the primary tumor or metastasis. All patients included must have been treated with standard of care therapy based on disease characteristics. Patients with incomplete data or follow up were excluded (Supplemental Figure 1). Putative driver mutations were alterations defined according to the commercial test or through the National Institute of Health’s ClinVar database. Variants of unknown significance (VUS) or alterations not listed in ClinVar were not considered pathogenic mutations. Specific pathways of interest were defined as follows: DNA double-strand break (DDSB) repair genes: *ATM, ATR, ATRX, BRCA1, BRCA2, BRIP1, CHEK2, FANCA, FANCC, FANCG, FANCF, FANCL, MRE11;* Wnt pathway genes: *CTNNB1, APC, and RNF43*; cell cycle genes: *CCND1-3, CCNE1, CDKN1B, CDKN2A, CDKN2B, CDK4, CDK6, RB1*; and PIK3/AKT/mTOR pathway genes: *AKT1, AKT2, PIK3R1, PIK3CA, PIK3CB, PIK3C2B, PIK3C2G, PTEN, MTOR, TSC1, TSC2* (Supplemental Figure 2).

Patients were defined as having either oligometastatic (≤5 lesions) or polymetastatic (≥6 lesions) using staging computed tomography (CT), positron emission tomography (PET)/CT, and MRI. Pleural disease was designated as low volume. Available follow-up data from serial physical examination and imaging were obtained; all follow-up procedures were in accordance with National Comprehensive Cancer Network guidelines and conducted in a regimented fashion by a multidisciplinary group of oncologists.

Baseline characteristics and frequency of putative driver mutations within each disease volume category were compared using a Chi-Square Test for Independence or Fisher’s Exact Test for categorical variables based on expected cell counts and Wilcoxon rank sum test for continuous variables. Median overall survival (mOS) was calculated from time of metastatic disease to death using the Kaplan Meier method, stratified by disease volume or mutation, and compared with the log-rank test. Pairwise tests were additionally performed to compare mOS between location of metastasis. Multivariable Cox regression (MVA) analyses were conducted for overall survival. Variables included in the MVA were those *a priori* predicted to be associated with outcomes. Fisher’s exact test was used for the calculation of statistical significance in DAVID analysis. Adjustments for multiple comparisons were made by controlling the false discovery rate (FDR) at the nominal 0.05 level using the Benjamini-Hochberg procedure. All analyses were conducted using R.

## Results

### Clinical Characteristics of Oligo - and Polymetastasis

A total of 406 patients were included (243 with oligometastasis and 163 with polymetastasis). Clinical characteristics are shown in Table 1. Polymetastasis was associated with more aggressive disease features such as higher incidence of nodal disease (72.3% vs. 59.2%, *P* = .03) and *de novo* metastasis (75.3% vs. 64.2%, *P <* .001). The number of organs involved were as follows: Oligometastatic - 1 site-65.4%; 2 sites-24.3%; 3 sites-8.2%; 4 sites-1.2%; 5 sites-0.8%; Polymetastatic: 1 site-26.4%; 2 sites-27%; 3 sites-21.5%; 4 sites-15.3%; 5 sites-6.1%; 6 sites-2.5%; 8 sites-0.6%; ten sites-0.6%

**Figure 1:**
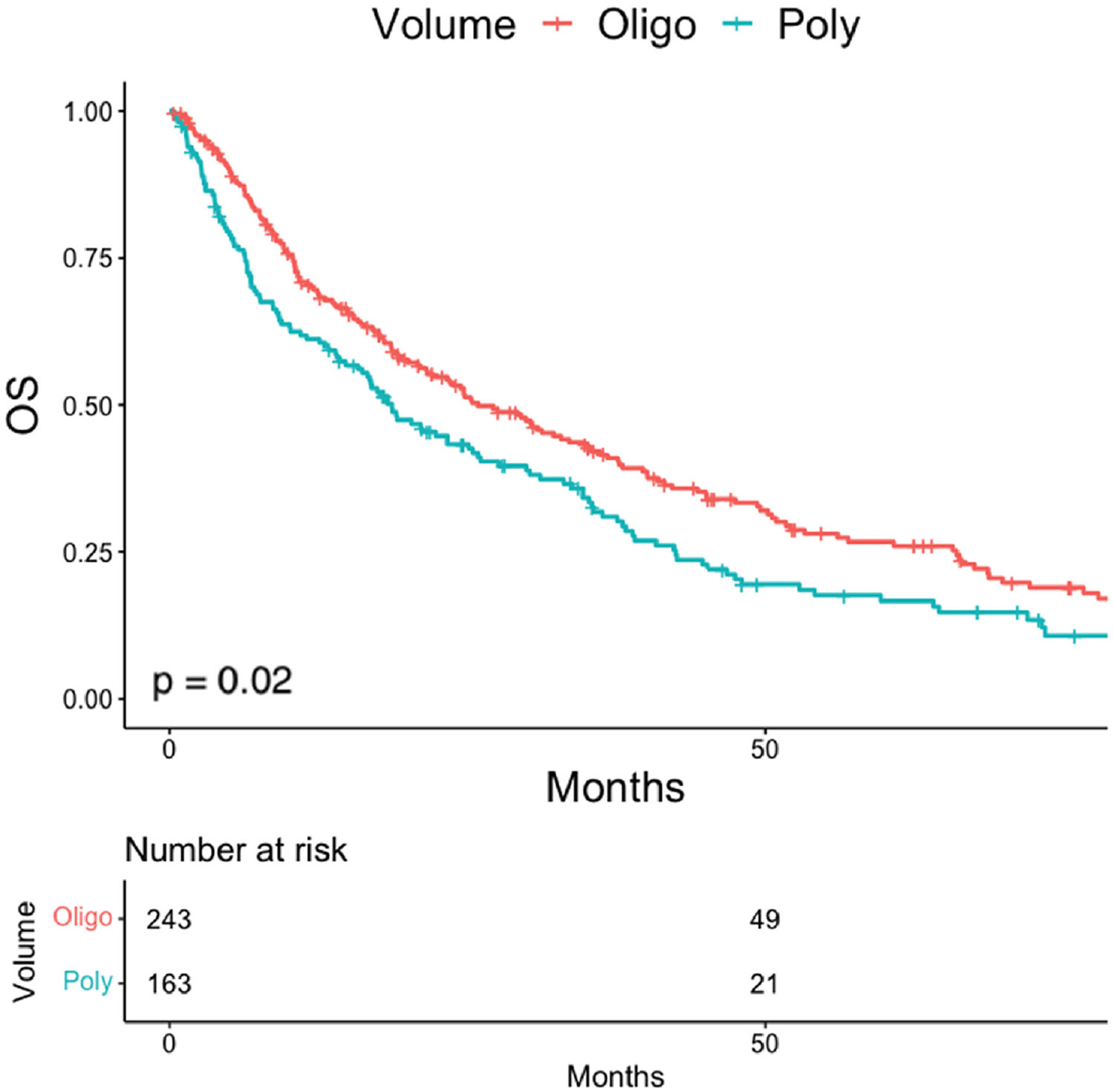
Overall survival stratified by disease volume.

Oligometastasis was associated with improved mOS (25.9 months, 95% CI, 21.6 months-35.1 months) when compared to polymetastasis (18.7 months, 95% CI, 14.8 months-25.9 months, *P* = .02, Figure 1). Patients with targetable mutations had improved survival (median overall survival 42.6 months vs. 16 months, *P <* .001). The prognostic benefit of oligometastasis remained when looking at patients without targetable mutations (19.5 months, 95% CI, 15.8-25.4 months versus 8.7 months, 95% CI, 6.515.8 months, *P* = .003). Location of metastasis did not impact median OS in patients with oligometastasis (pleura: 32.2 months, lymph nodes: 52.1 months, lung: 30.9 months, bone: 17.6 months, visceral: 23.8 months, Supplemental Figure 3, pairwise comparison in Supplemental Table 1). In multivariable analysis (Table 2), oligometastasis remained associated with lower risk of death (HR 0.60, 95% CI, 0.46-0.76, *P <* .001). Other factors associated with OS were presence of a targetable mutation (HR 0.39, 95% CI, 0.29-0.52, *P <* .001) and age (HR 1.02, 95% CI, 1.01-1.03, *P*= .002, Table 2).

### Genetic Features of Oligo - and Polymetastasis

**Figure 2:**
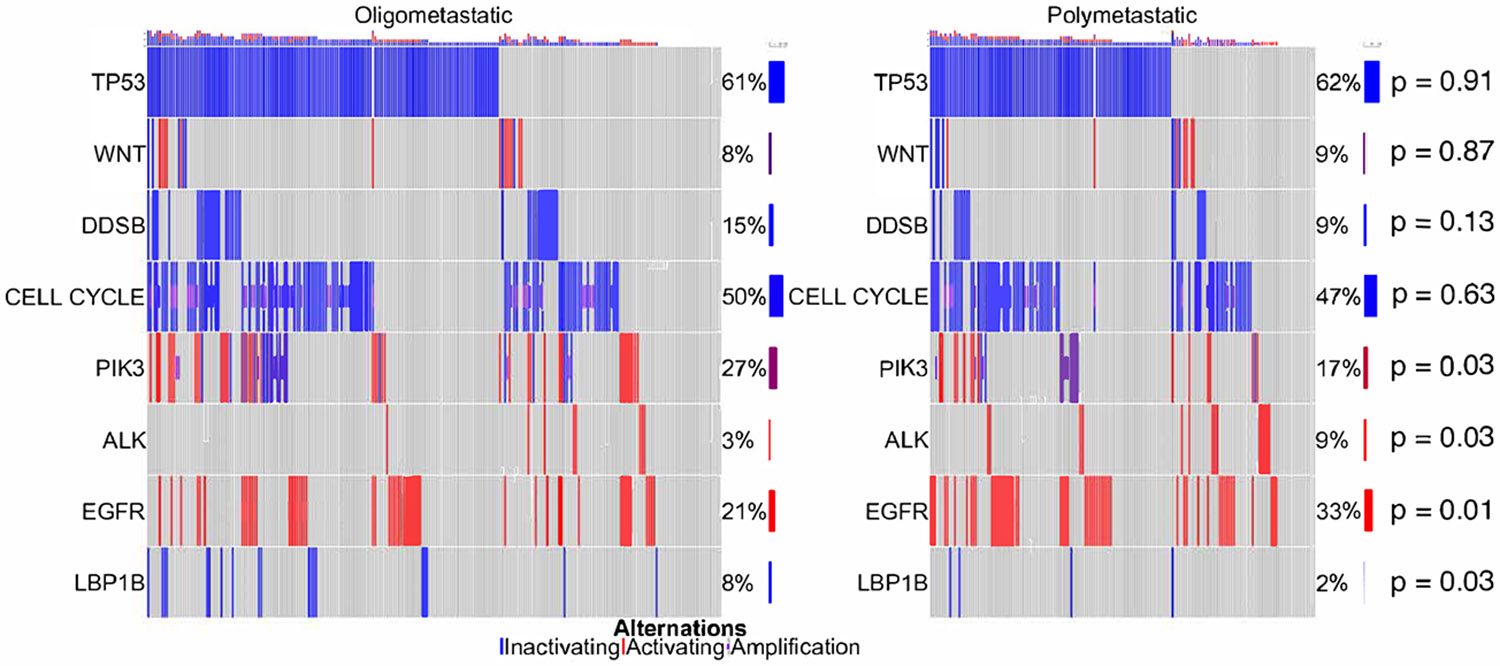
Mutation status stratified by disease volume.

Genetic features differed between oligometastatic and polymetastatic NSCLC (Figure 2). Oligometastatic patients tended to have higher incidence of alterations in *PI3K* pathway genes (24.3% vs. 14.7%, *P* = .03) and *LRPB1* (7.8% vs. 2.5%, *P* = .03). In contrast, polymetastatic patients tended to have higher rates of alterations in *EGFR* (33.1% vs. 21.4%, *P* = .01) and *ALK* (8.6% vs. 3.3%, *P* = .03). The most common alterations in oligometastasis and polymetastasis are shown in Table 3. *TP53* was the most frequent mutation in both groups. Other frequent alterations in oligo- and polymetastasis included *EGFR, CDKN2A, KRAS, CDKN2B, NKX2-1, Rb1, NFKBIA, STK11, MYC, and PIK3CA.* Alterations commonly seen within oligometastasis but not polymetastasis included *PTEN, RICTOR, ARID1A*, and *LRBP1* while those frequently seen in polymetastasis but not oligometastasis included *ALK, ERBB2, MET*, and *MDM2*. Several alterations were only seen within oligometastasis (Supplemental Table 2) including mutations within the FANC core protein complex (*FANCA, FANCF, and FANCL*) and fibroblast growth factors (*FGF6, FGF12, FGF23, FGFR2*, and *FGFR4*).

On DAVID analysis, oligo-and polymetastatic tumors demonstrated differences in pathway enrichment. Oligometastatic tumors demonstrated enrichment in signaling response to DNA damage, ATM signaling, and NOTCH pathway signaling. Polymetastatic tumors demonstrated enrichment in pathways related to motility and epithelial mesenchymal transition (EMT) including WNT pathway signaling, HIF-1 signaling, TGF beta signaling, NF kappa B, VEGF signaling, and lamellipodium function (Figure 3A and B).

### Prognostic Biomarkers in Oligo - and Polymetastasis

Several alterations were prognostic for survival within oligometastasis. Patients with *EGFR* (50.9 months, [95% CI, 45 months-NR] vs. 21.6 months, [95% CI, 17.2 months-29.5 months], *P <* .0001) and *ALK* (111 months, [95% CI, 82.3 months-NR] vs. 24.8 months, [95% CI 19.5 months.−33.5 months], *P* = .01) mutations experienced significantly prolonged mOS (Supplemental Figure 4A-B). *EGFR* and *ALK* were similarly prognostic in polymetastatic disease (Supplemental Figure 5A-B). *TP53* (mOS 17 months, [95% CI, 10.1 months-23.2 months] vs. 25.9 months, [95% CI, 16.8 months-45.1 months], *P* = .01) and *KRAS* (mOS 10.2 months, [95% CI, 5.0 months-25.5 months] vs. 21.2 months, [95% CI, 17.4 months-31.1 months], *P* = .06) mutations were prognostic in polymetastatic but less so in oligometastatic disease (*TP53* mOS: 21.7 months, [95% CI, 18 months-32.2 months] vs. 33.5 months, [95% CI, 24.8 months vs. 50.2 months], *P* = .2; KRAS mOS: 19 months, [95% CI, 12.1 months-40.1 months] vs. 27.5 months, [95% CI, 23.2 months-37.7 months], *P* = .19, Supplemental Figure 6A-D).

## Discussion

In this study we aimed to understand clinical and genetic differences in NSCLC based on disease volume. Polymetastasis was associated with more aggressive disease and inferior mOS when compared to oligometastasis. Additionally, differences were noted between oligo- and polymetastasis with regards to genetic make-up. Polymetastatic tumors tending to have higher incidence of alterations in *EGFR* and *ALK* while oligometastatic disease tended to have higher incidence in alterations in *LRP1B* and the *PIK3* pathway. Additionally, pathways within cell motility and EMT appeared to be significantly enriched in polymetastatic tumors compared to oligometastatic tumors.

In this study we identified oligometastatic disease associated with better prognosis compared to polymetastatic disease in NSCLC, in line with other studies.^14^ The range of outcomes in the spectrum of metastasis, especially the favorable responses that can be seen in oligometastasis, has led to interests in intensifying treatment for patients with low volume metastatic disease. Several prospective randomized trials now demonstrate integration of consolidative local therapies in de novo oligometastatic NSCLC improve outcomes,^4,5,7^ including Gomez et al.^4^ where mOS was improved from 17 months to 41.2 months with consolidative radiation. Additional prospective trials in metachronous oligometastatic prostate cancer^3,8,11^ as well as a mixture of histologies^9,10^ demonstrate similar benefits with local consolidative therapies. Future prospective trial will now need to better identify patients who might benefit from these interventions, and in contrast develop novel paradigms for those who do not.

Current methods for defining and selecting patients for oligometastatic paradigms revolve around lesion enumeration, which is likely a surrogate for a disease biology with an attenuated metastatic phenotype potentially amenable to local consolidative therapy. However, despite this, lesion number remains a rudimentary approximation of disease biology, and many patients with oligometastasis treated with local consolidative therapy might attain relatively minor benefit. Conversely, there are also likely patients with larger volume disease with biologies that would be amenable to metastasis directed therapy, though not candidates based on lesion number alone. Thus, additional biomarkers are necessary to better classify patients, for which genomics holds promise. This has been explored within oligometastatic prostate cancer where alterations within DNA damage response genes, especially *TP53*, might better identify disease biology than disease volume, and these alterations are enriched in polymetastatic vs. oligometastatic cohorts.^3, 12^ In line with these findings, patients with metastatic disease seen on enhanced, but not conventional imaging, are also less likely to harbor mutations in *TP53.*^15^ Relatively less is known about the genetic make-up of NSCLC by disease volume. In our study there appeared to be differences in genetic composition between disease volume groups with *LRP1B* alterations noted to be more common in the oligometastatic cohort. *LRP1B* has been showed in several studies to be associated with a favorable prognosis possibly explaining some of the biological behavior of the oligometastatic cohort.^16-18^ Conversely *EGFR* and *ALK* mutation were seen with higher incidence in the polymetastatic cohort. *EGFR* gains and *ALK* mutations are enriched in metastatic sites when compared to the primary tumor, suggesting their importance in cancer invasion and metastasis.^19^ Interestingly, neither *PIK3/Akt/mTOR* pathway alterations, which were noted to be higher within the oligometastatic cohort, nor *LRP1B* mutations were enriched in metastatic sites. Additionally, various STK11 mutations have different functional impacts on cancer invasion and metastasis.^20^ Future work will have to build on these findings to try and better biologically define disease volume in metastatic NSCLC.

While *EGFR* and *ALK* mutations seem to be associated with higher volume of disease, in those who do receive local consolidative therapy, *EGFR* alterations appear to be associated with better prognosis.^21^ In our cohort *EGFR* alterations were also associated with better prognosis. These findings are likely due to the ability to use tyrosine kinase inhibitors to target these alterations. The more aggressive disease biology and poor prognosis of polymetastasis is likely additionally explained by other differences in genetic make-up. When examining the larger genomic landscape using DAVID analysis, we noted polymetastatic tumors tended to have enrichment in pathways such as WNT pathway signaling, HIF-1 signaling, TGF beta signaling, NF kappa B, VEGF signalling, and lamellipodium function which are associated with cell motility or EMT.^22-25^ EMT is well known to enhance cancer motility, invasion, and metastasis in preclinical models through loss of epithelial markers and ability to migrate^26^ which could in part explain dissemination seen in polymetastatic tumors. In the clinical setting, WNT alterations are associated with more advanced sites of metastatic disease in castration sensitive prostate cancer with higher incidence of visceral as compared to pelvic lymph node metastases.^12^ These findings might give a biological rationale for phenotypic behavior of metastases, however additional preclinical models are needed which in turn might allow for better discrimination of metastases behaving in a loco-regional manner. Additionally, these pathways might be able to be targeted with greater success in the future when combined with MDT.

This study has several limitations. First, it is a retrospective cohort and endpoints were not prospectively defined. Thus, this study is susceptible to the caveats of a retrospective study. Multivariable analysis was performed to attempt to mitigate possible confounders but prospective validation will still be necessary. Some possible confounders, such as higher ECOG group could be limited due to small numbers. Additionally, genes were limited to those on the panel, which are commonly mutated with malignant processes. Furthermore, patients were treated with various first line systemic agents such as chemotherapy, immunotherapy, and targeted agents. Thus, although all patients were treated per standard of care therapies, the heterogeneity of treatment can introduce bias. Finally, there is a skew with higher percentage of patient’s adenocarcinoma in the analysis as these patients are more likely to undergo NGS as standard of care therapy and thus further insight is needed for patients with squamous cell carcinoma.

## Conclusion

In conclusion, oligo– and polymetastatic NSCLC have different clinical and genomic features. Oligometastatic tumors have a better prognosis and appears to be associated with higher incidence of alterations in *PI3K* pathway genes and *LRPB1*, while polymetastatic tumors have poorer prognosis, higher incidence of *EGFR* and *ALK* mutations, and have enrichment in pathways such as WNT pathway signaling, HIF-1 signaling, TGF beta signaling, NF kappa B, VEGF signaling, and lamellipodium function which are associated with cell motility or EMT. These findings can hopefully be expanded upon to develop more biological definitions of metastatic volume to help in prognostication and the treatment decision making process.

### Clinical practice points

- Oligometastasis is increasingly being managed with local therapies
- However, definitions of this state are rudimentary.
- Here we show underlying genomic differences based on disease volume in patients with non-small cell lung cancer.
- These findings can help to refine definitions of oligometastasis to possibly allow for better personalization of treatment.

## Supplementary Material

Supp Fig 3 Supplemental Figure 3 Overall survival stratified by metastasis location.

Supp Fig 2 Supplemental Figure 2 Genes of interest grouped by pathways for analysis.

Supp Fig 4A Supplemental Figure 4A Overall survival for oligometastatic patients stratified by *EGFR* status.

Supp Fig 5B Supplemental Figure 5B Overall survival for polymetastatic patients stratified by *ALK* status.

Supp Fig 4B Supplemental Figure 4B Overall survival for oligometastatic patients stratified by *ALK* status.

Supp Fig 5A Supplemental Figure 5A Overall survival for polymetastatic patients stratified by *EGFR* status.

Supp Fig 6B Supplemental Figure 6B Overall survival for polymetastatic patients stratified by *KRAS* status.

Supp Fig 6A Supplemental Figure 6A Overall survival for polymetastatic patients stratified by *TP53* status.

Supp Fig 6C Supplemental Figure 6C Overall survival for oligometastatic patients stratified by *TP53* status.

Supp Table 1 Suppemental Table 1 Pairwise comparisons for outcomes based on metastasis location

Supp Fig 6D Supplemental Figure 6D Overall survival for oligometastatic patients stratified by *KRAS* status.

Supp Table 2 Suppemental Table 2 Genetic alterations only seen in I

Supplementary 1 Supplemental Figure 1 Consort diagram for patientin clusion in study.

## Figures and Tables

**Figure 3A:**
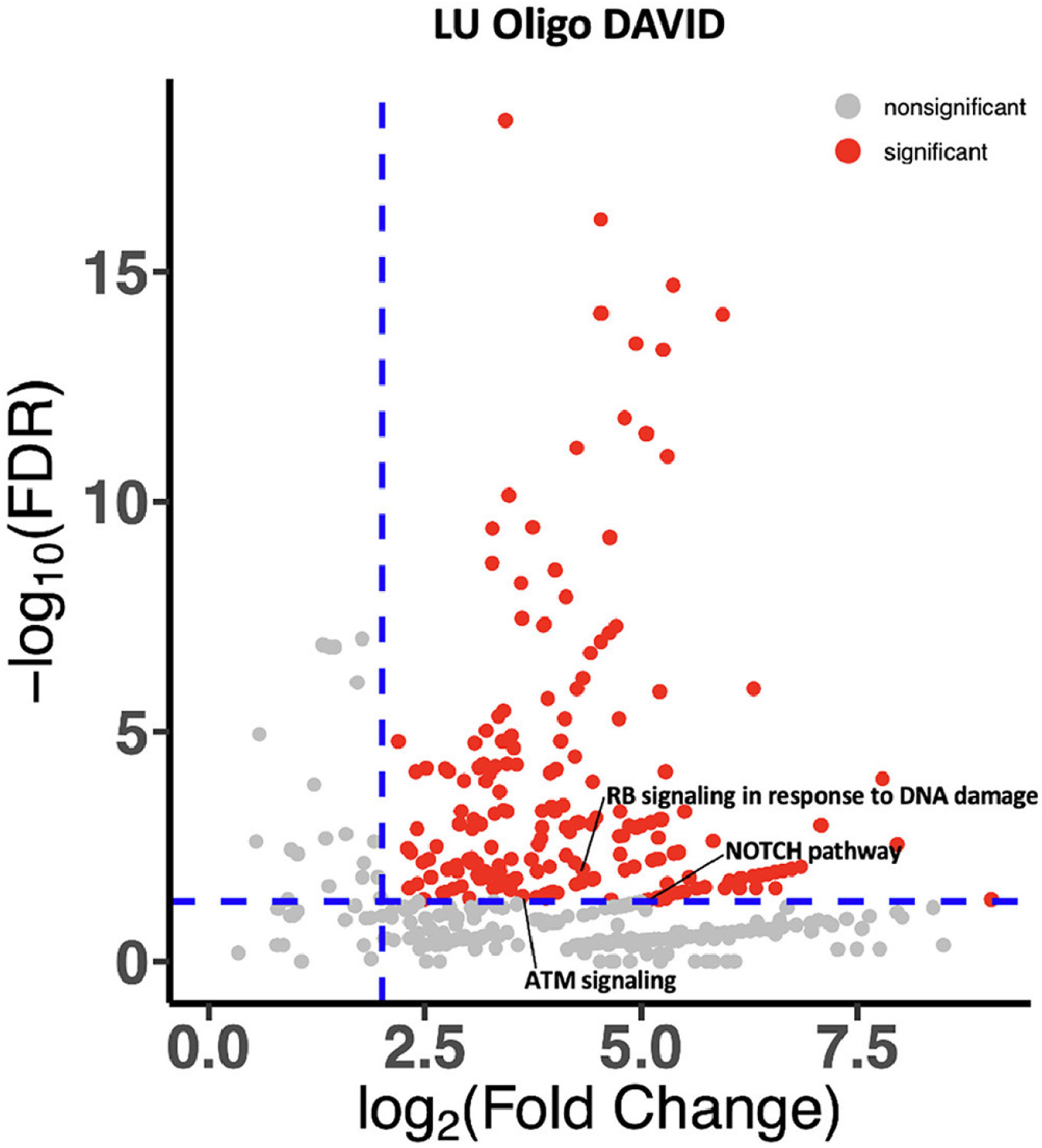
DAVID analysis for oligometastatic patients.

**Figure 3B:**
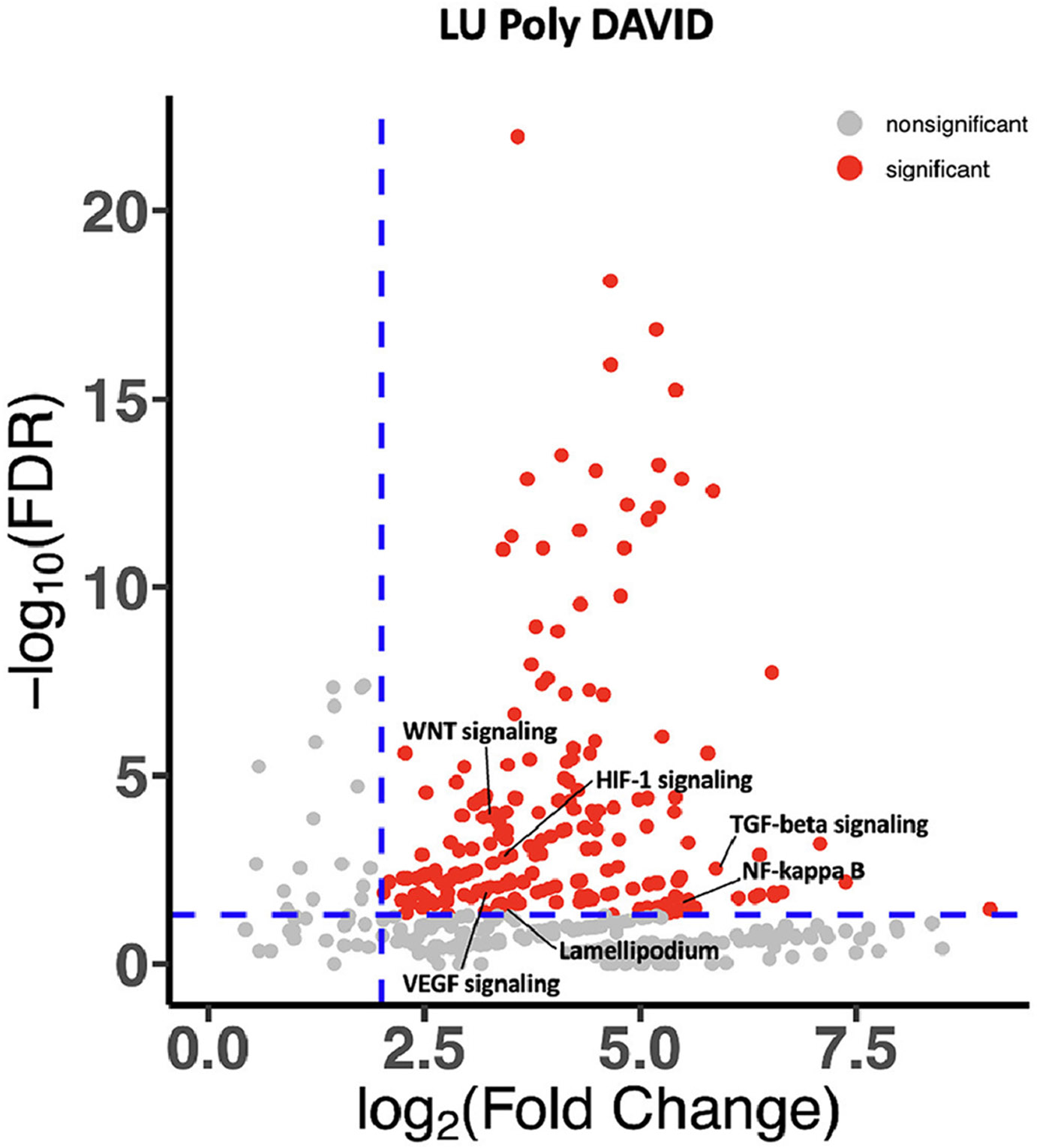
DAVID analysis for polymetastatic patients.

**Table 1.** Baseline characteristics stratified by disease volume

Variable	Oligo	Poly	p value
Age (years)	64.2	64.6	0.83
Gender			0.74
Male	114 (46.9%)	80 (49.1%)	
Female	129 (53.1%)	83 (50.9%)	
T stage			0.06
Tx	13 (5.4%)	7 (4.4%)	
T1	69 (28.7%)	26 (16.4%)	
T2	55 (22.9%)	44 (27.7%)	
T3	37 (15.4%)	32 (20.1%)	
T4	66 (27.5%)	50 (31.4%)	
N stage			0.03
N0	98 (40.8%)	44 (27.7%)	
N1	17 (7.1%)	8 (5%)	
N2	92 (38.3%)	78 (49.1%)	
N3	33 (13.8%)	29 (18.2%)	
M stage			<0.001
M0	87 (35.8%)	40 (24.7%)	
M1	156 (64.2%)	122 (75.3%)	
Histology			0.002
SCC	40 (16.6%)	10 (6.1%)	
Adeno	199 (82.6%)	148 (90.8%)	
Poorly diff	2 (0.8%)	5 (3.1%)	
Treatment			
Definitive local	87 (35.8%)	41 (25.2%)	0.03
Systemic	156 (64.2%)	122 (74.8%)	
ECOG			0.12
0	64 (26.2%)	48 (29.4%)	
1	118 (48.6%)	95 (58.2%)	
2	43 (17.7%)	8 (5%)	
3	18 (7.4%)	10 (6.1%)	
4	2 (0.8%)	2 (1.2%)	
TPS			0.44
<1%	81 (33.3%)	76 (46.6%)	
1-49%	92 (37.9%)	58 (40.6%)	
>50%	70 28.8)	29 (20.3%)	
Systemic therapy			0.03
Targeted	60 (24.7%)	59 (41.3%)	
Chemo/Immuno	183 (75.3%)	84 (58.7%)	

SCC: squamous cell carcinoma; Adeno: adenocarcinoma; poorly diff: poorly differentiated; chemo: chemotherapy; immune: immunotherapy; TPS: tumor proportion score; ECOG: Eastern Cooperative Oncology Group

**Table 2.** Multivariable analysis for variables associated with overall survival

	HR (95% CI)	p value
Oligometastatic	0.60 (0.47 – 0.76)	<0.001
Targetable mutation	0.39 (0.29 – 0.52)	<0.001
M1 (vs M0)	1.17 (0.90 – 1.53)	0.23
N stage (vs N0)		
N1	1.32 (0.83 – 2.22)	0.22
N2	1.33 (1.02 – 1.74)	0.04
N3	0.77 (0.53 – 1.14)	0.20
T stage (vs T1)		
Tx	0.51 (0.25 – 1.02)	0.06
T2	1.36 (0.98 – 1.89)	0.06
T3	1.01 (0.69 – 1.47)	0.96
T4	0.94 (0.67 – 1.30)	0.69
Age	1.02 (1.01 – 1.03)	0.002
Histology (vs Adeno)		
SCC	1.05 (0.74 – 1.49)	0.78
Poorly diff	3.55 (1.51 – 8.37)	0.004

SCC: squamous cell carcinoma; Adeno: adenocarcinoma; poorly diff: poorly differentiated

**Table 3.** Most common alteration based on disease volume

Gene	Oligometastatic (n=243)	Polymetastatic (n=163)
*TP53*	60.9%	62%
*CDK2NA*	28%	30.7%
*KRAS*	28%	21.5%
*EGFR*	21.4%	33.1%
*CDKN2B*	19.8%	19%
*STK11*	17.3%	9.8%
*PIK3CA*	11.5%	7.4%
*MYC*	10.3%	8.6%
*PTEN*	8.6%	
*NKX2-1*	8.6%	13.5%
*Rb1*	8.2%	11%
*NFKBIA*	8.2%	10.4%
*RICTOR*	8.2%	
*ARID1A*	7.8%	
*LRBP1*	7.8%	
*ALK*		8.6%
*ERBB2*		8.0%
*MET*		8.0%
*MDM2*		7.4%
