# Supplementary figures and images for: Clinical and Genomic Features and Prognostic Biomarkers of Oligometastatic Nonsmall Cell Lung Cancer
Source: Clin Lung Cancer. Author manuscript; Available in PMC 2026 Apr 15. (PMC13080795; doi:10.1016/j.cllc.2025.07.010)

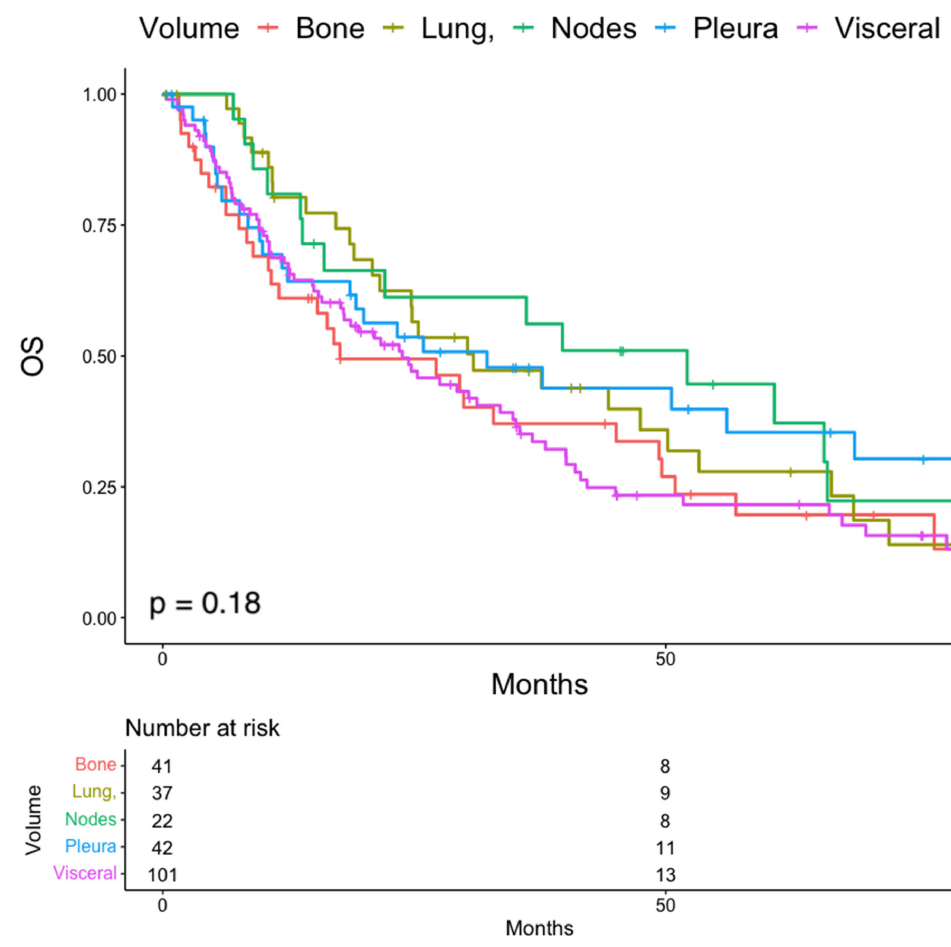

Supplement: Supp Fig 3 — Supplemental Figure 3 Overall survival stratified by metastasis location. [file NIHMS2147508-supplement-Supp_Fig_3.pdf]

# Dysregulated genes and pathways of interest

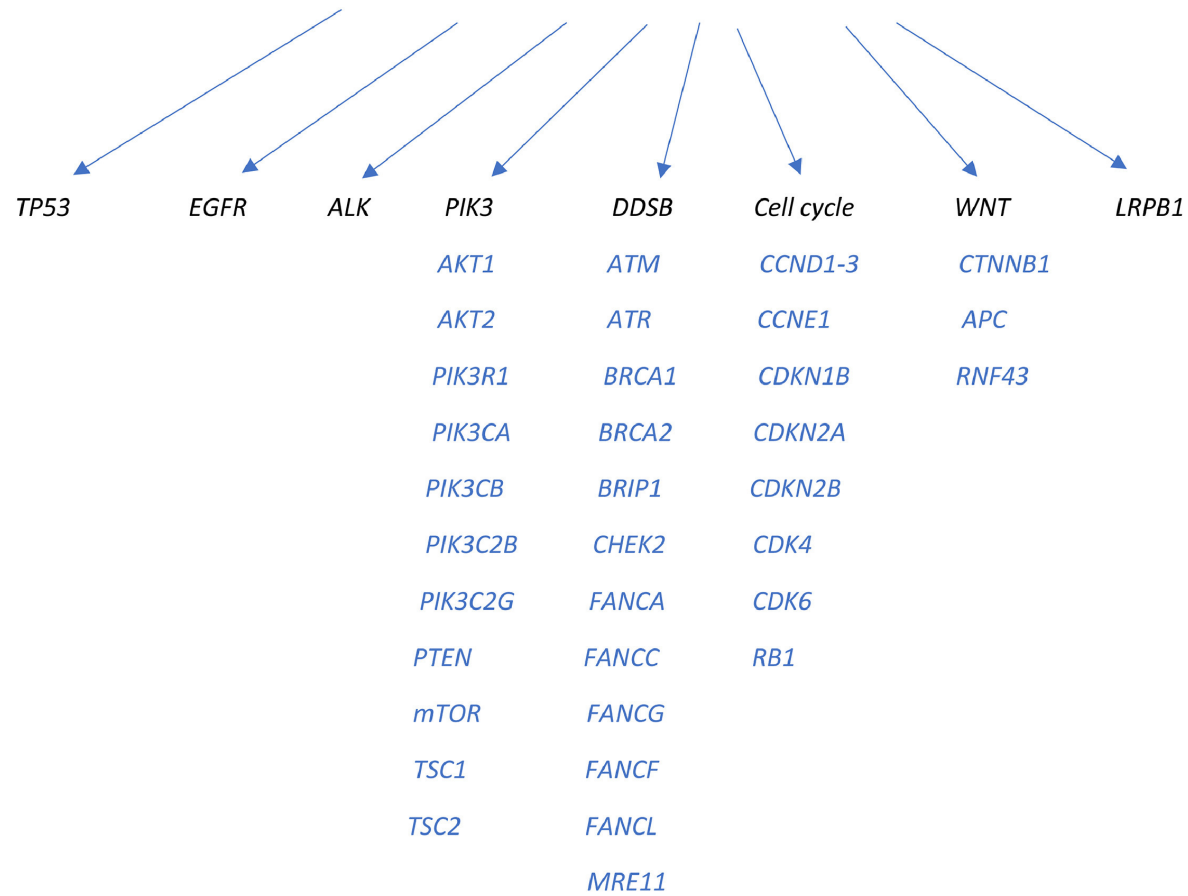

Supplement: Supp Fig 2 — Supplemental Figure 2 Genes of interest grouped by pathways for analysis. [file NIHMS2147508-supplement-Supp_Fig_2.pdf]

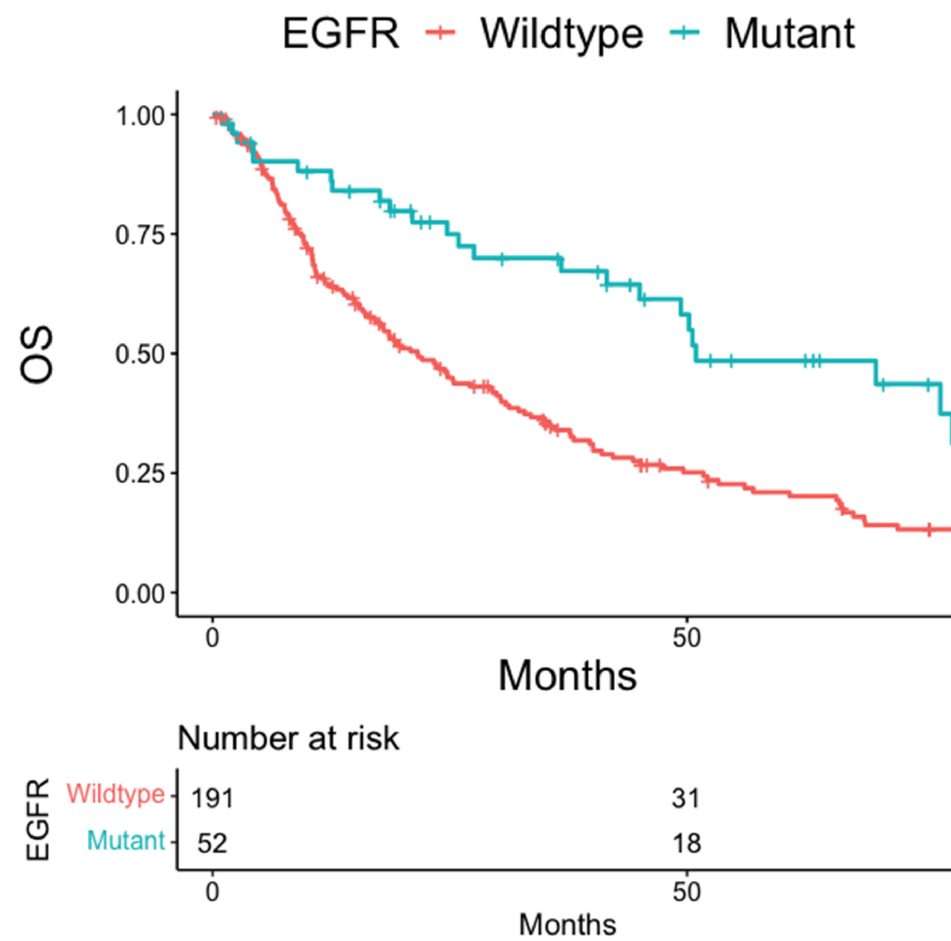

Supplement: Supp Fig 4A — Supplemental Figure 4A Overall survival for oligometastatic patients stratified by EGFR status. [file NIHMS2147508-supplement-Supp_Fig_4A.pdf]

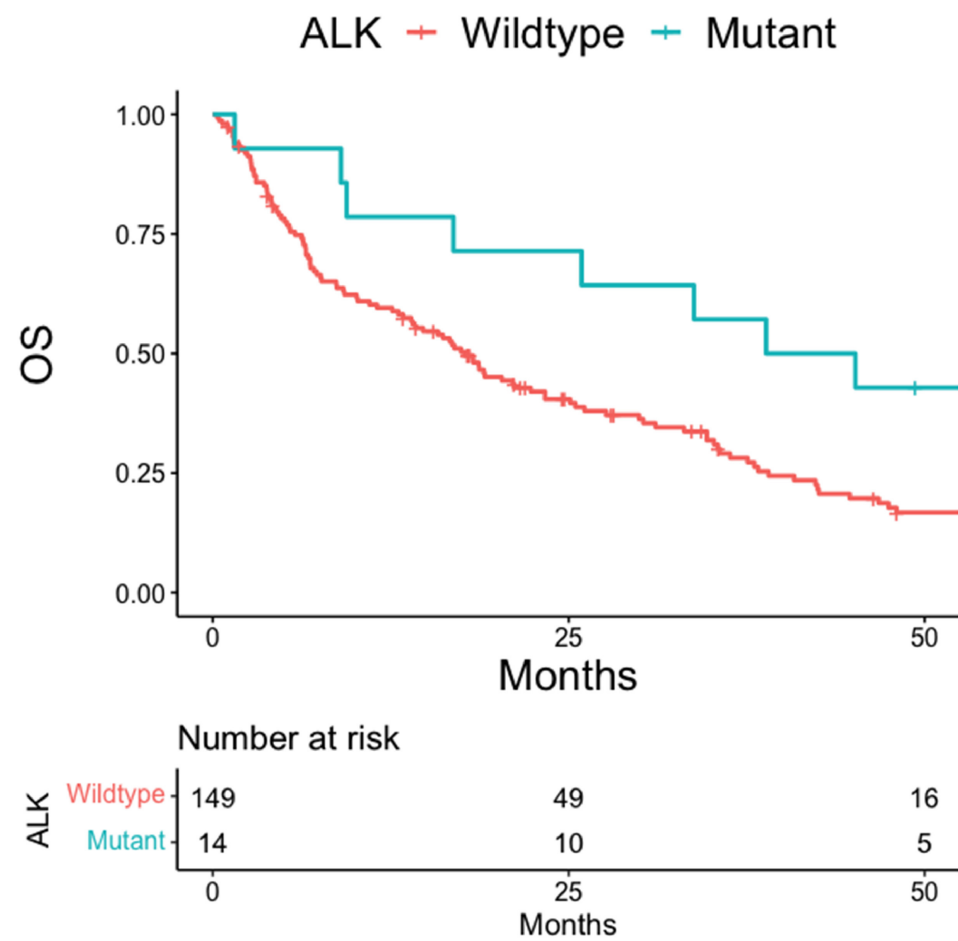

Supplement: Supp Fig 5B — Supplemental Figure 5B Overall survival for polymetastatic patients stratified by ALK status. [file NIHMS2147508-supplement-Supp_Fig_5B.pdf]

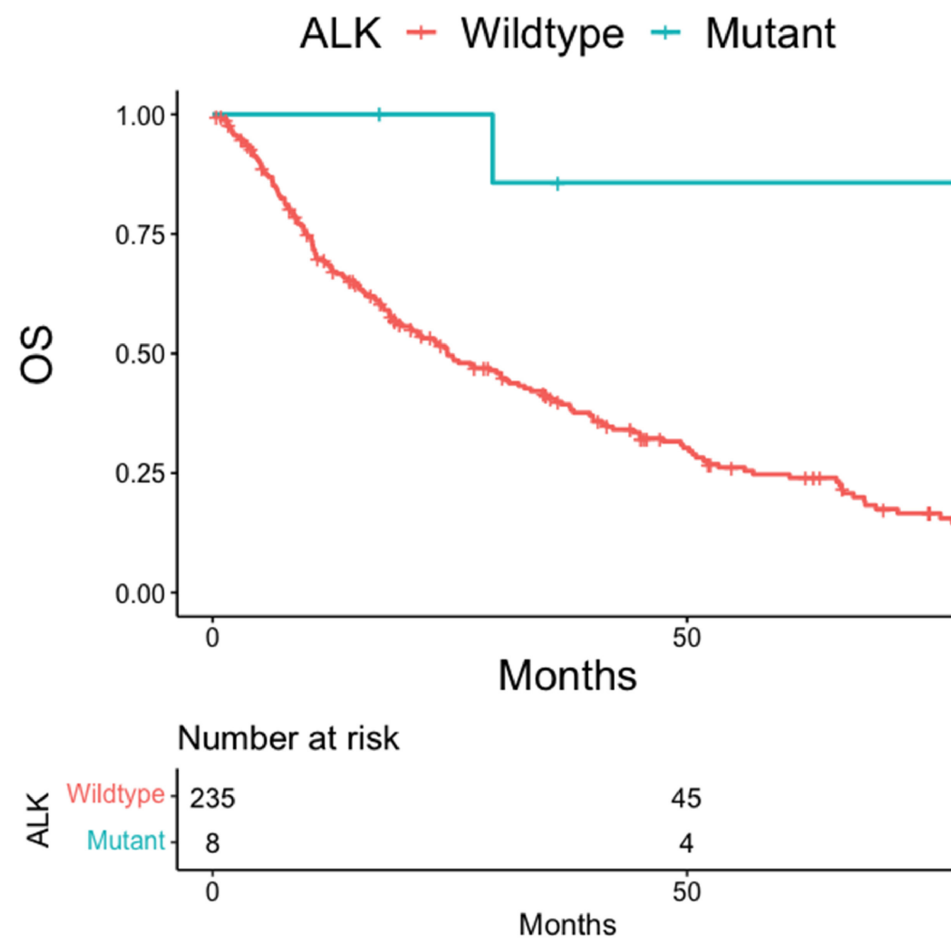

Supplement: Supp Fig 4B — Supplemental Figure 4B Overall survival for oligometastatic patients stratified by ALK status. [file NIHMS2147508-supplement-Supp_Fig_4B.pdf]

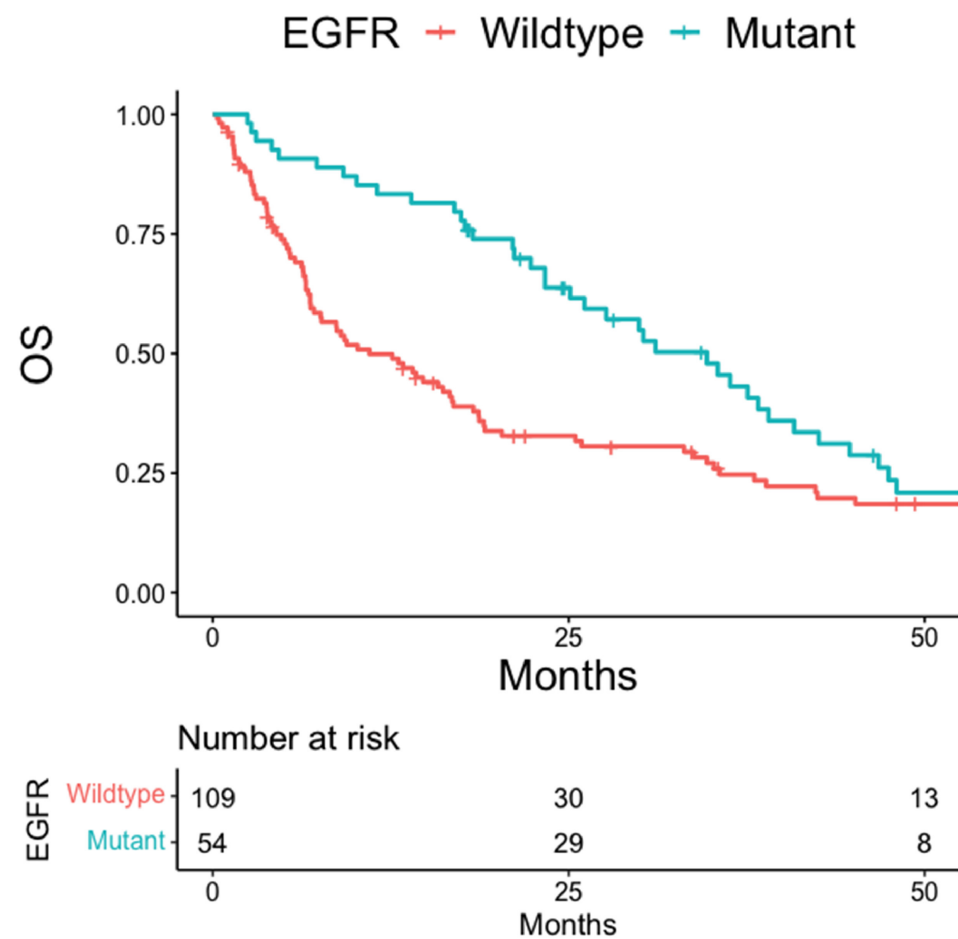

Supplement: Supp Fig 5A — Supplemental Figure 5A Overall survival for polymetastatic patients stratified by EGFR status. [file NIHMS2147508-supplement-Supp_Fig_5A.pdf]

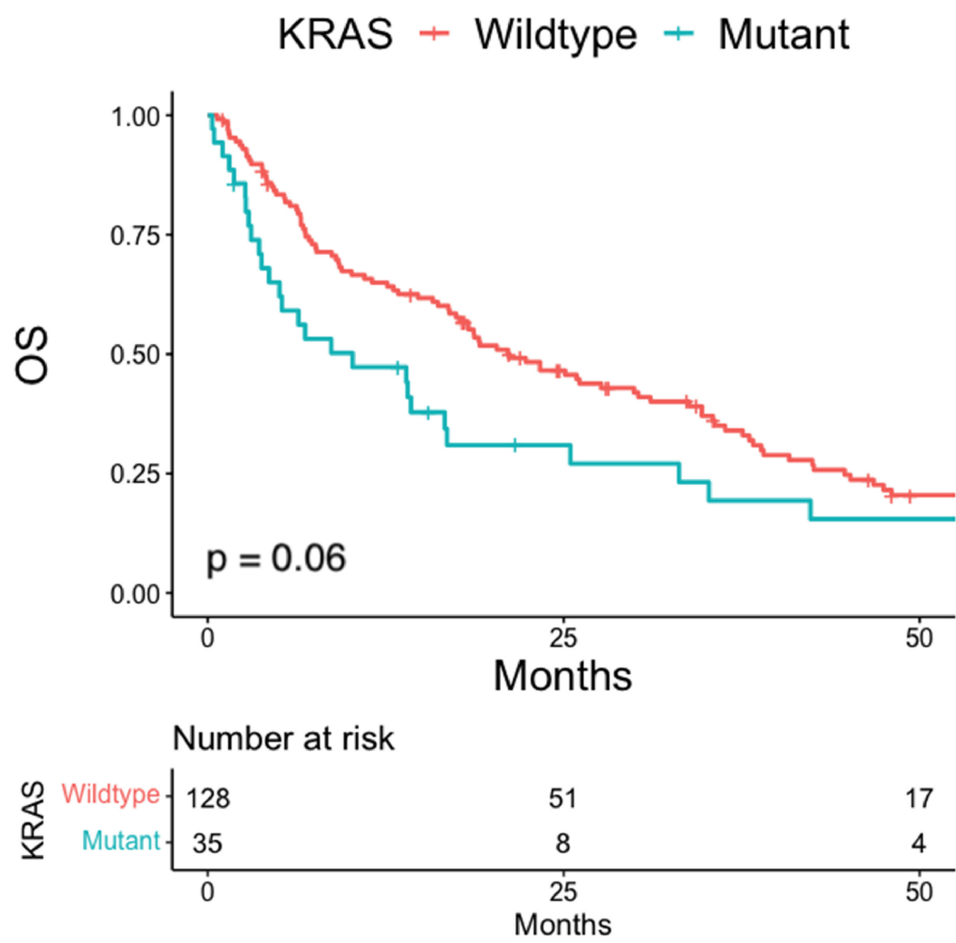

Supplement: Supp Fig 6B — Supplemental Figure 6B Overall survival for polymetastatic patients stratified by KRAS status. [file NIHMS2147508-supplement-Supp_Fig_6B.pdf]

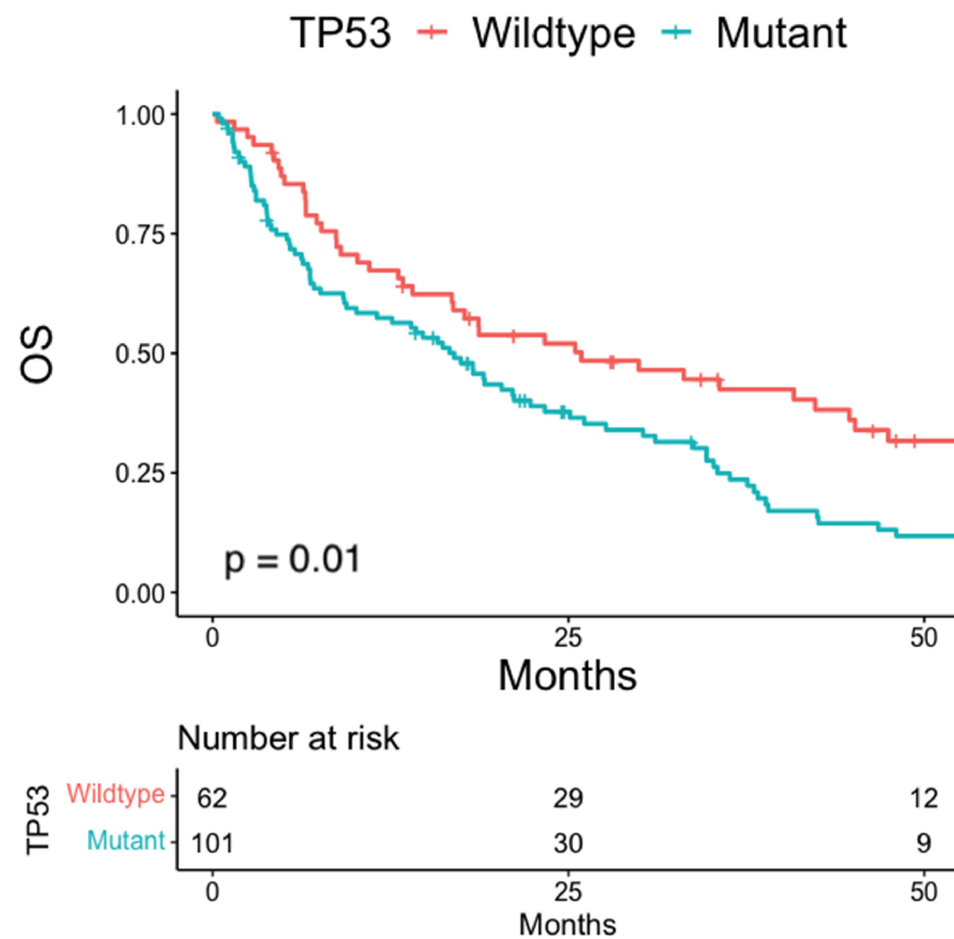

Supplement: Supp Fig 6A — Supplemental Figure 6A Overall survival for polymetastatic patients stratified by TP53 status. [file NIHMS2147508-supplement-Supp_Fig_6A.pdf]

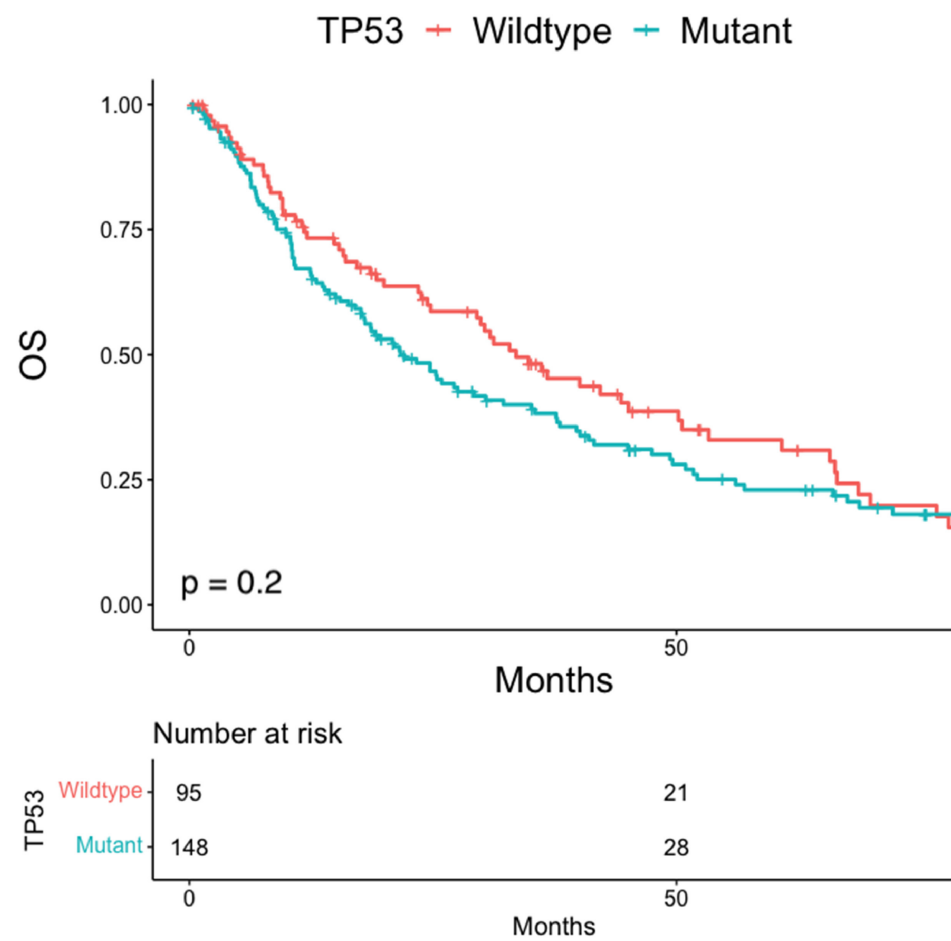

Supplement: Supp Fig 6C — Supplemental Figure 6C Overall survival for oligometastatic patients stratified by TP53 status. [file NIHMS2147508-supplement-Supp_Fig_6C.pdf]

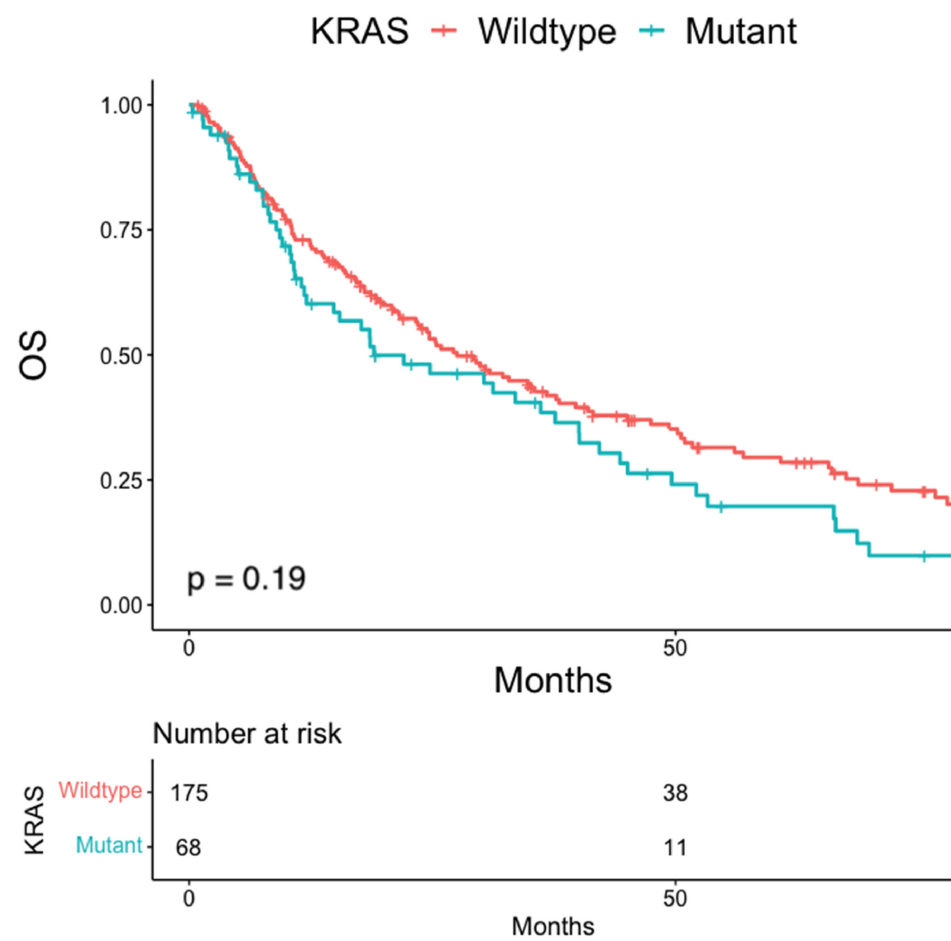

Supplement: Supp Fig 6D — Supplemental Figure 6D Overall survival for oligometastatic patients stratified by KRAS status. [file NIHMS2147508-supplement-Supp_Fig_6D.pdf]
