# Supplementary material for: Clinical and Genomic Features and Prognostic Biomarkers of Oligometastatic Nonsmall Cell Lung Cancer
Source: Clin Lung Cancer. Author manuscript; Available in PMC 2026 Apr 15. (PMC13080795; doi:10.1016/j.cllc.2025.07.010)
Supplement: Supp Table 1 — Suppemental Table 1 Pairwise comparisons for outcomes based on metastasis location [file NIHMS2147508-supplement-Supp_Table_1.pdf]

Supplemental table 1. Pairwise comparisons for outcomes based on metastasis location

Empty Cell	**p value**
**Bone vs. Lung**	0.52
**Bone vs. Node**	0.43
**Bone vs. Pleura**	0.43
**Bone vs. Viscera**	0.93
**Lung vs. Node**	0.79
**Lung vs. Pleura**	0.76
**Lung vs. Viscera**	0.43
**Node vs. Pleura**	0.93
**Node vs. Viscera**	0.43
**Pleura vs. Viscera**	0.43
