# Supplementary material for: Clinical and Genomic Features and Prognostic Biomarkers of Oligometastatic Nonsmall Cell Lung Cancer
Source: Clin Lung Cancer. Author manuscript; Available in PMC 2026 Apr 15. (PMC13080795; doi:10.1016/j.cllc.2025.07.010)
Supplement: Supp Table 2 — Suppemental Table 2 Genetic alterations only seen in I [file NIHMS2147508-supplement-Supp_Table_2.pdf]

Supplemental Table 2. Genetic alterations only seen in oligometastasis

**Gene**
FANCA
FANCF
FANCL
MRE11
CCND2
CDKN1B
MTOR
PIK3R1
ARID1B
BARD1
BAP1
BRD4
C27ORF39
CDH1
CDH2
CHEK1
CYLD
DAXXL

DICER1
ERBB4
FAM123B
FGF6
FGF12
FGFR2
FGFR4
FLT1
FLT3
FOXP1
IKZF1
IRS2
INPPB4
KDM6A
MAGI2
MAP2K1
MDM4
MITF
MYCL1

PDCD1LG2
PMS2
PTCH1
RAD50
RPTOR
RUNX1
SLIT2
SMARCB1
SPOP
STAT4
SUFU
TMEM30A
TNFAIP3
