# Supplementary material for: Clinical and Genomic Features and Prognostic Biomarkers of Oligometastatic Nonsmall Cell Lung Cancer
Source: Clin Lung Cancer. Author manuscript; Available in PMC 2026 Apr 15. (PMC13080795; doi:10.1016/j.cllc.2025.07.010)
Supplement: Supplementary 1 — Supplemental Figure 1 Consort diagram for patientin clusion in study. [file NIHMS2147508-supplement-Supplementary_1.pdf]

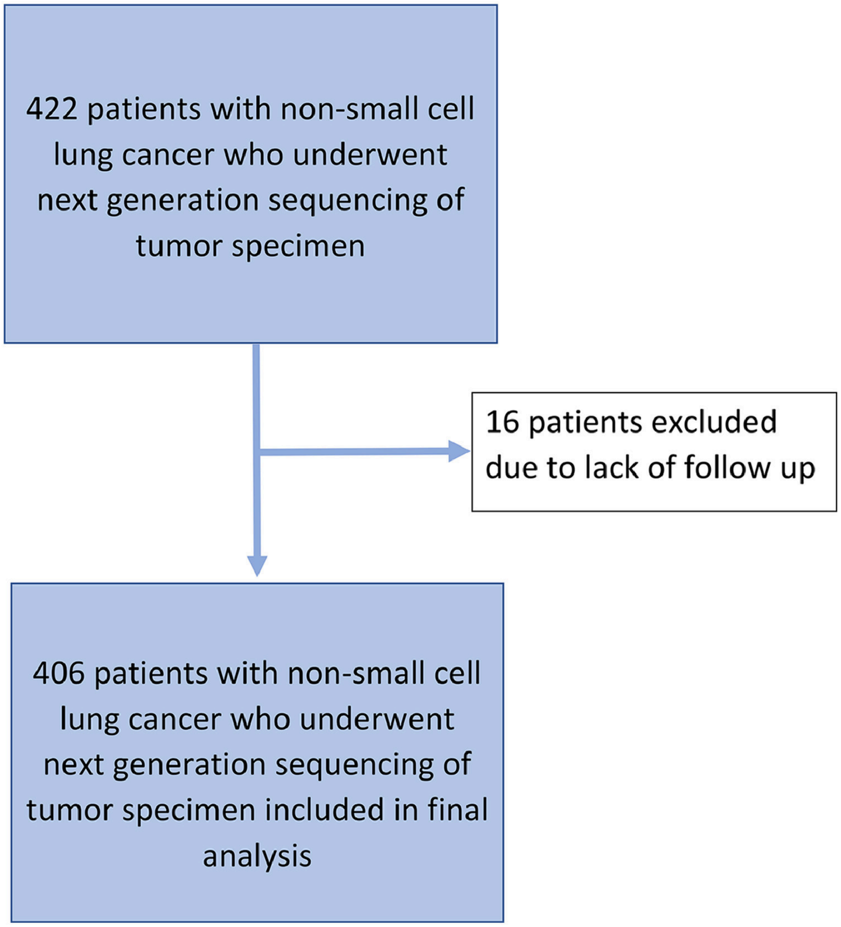
422 patients with non-small cell lung cancer who underwent next generation sequencing of tumor specimen
16 patients excluded due to lack of follow up
406 patients with non-small cell lung cancer who underwent next generation sequencing of tumor specimen included in final analysis
